# Congenital Ocular Motor Apraxia as the First Sign of Joubert Syndrome: A Case Report

**DOI:** 10.7759/cureus.103046

**Published:** 2026-02-05

**Authors:** Megumi Ito, Chihiro Koiwa, Takashi Negishi, Daisuke Sawada, Shintaro Nakao

**Affiliations:** 1 Department of Ophthalmology, Juntendo University, Tokyo, JPN; 2 Division of Child Neurology, Chiba Children's Hospital, Chiba, JPN; 3 Department of Pediatrics, Chiba University Graduate School of Medicine, Chiba, JPN

**Keywords:** brain mri, congenital ocular motor apraxia (coma), developmental delay, joubert syndrome (js), molar tooth sign

## Abstract

Congenital ocular motor apraxia (COMA) is characterized by an inability to initiate voluntary horizontal saccades, often leading patients to compensate with characteristic head thrusts. While COMA can sometimes present as an isolated ocular finding, it is frequently a manifestation of broader neurodevelopmental disorders. We report the case of a nine-month-old female infant presenting with developmental delay and abnormal eye movements. Examination revealed marked motor developmental delay, hypertonia, truncal arching, and poor head control. Notably, oculomotor apraxia requiring compensatory head thrusts was observed, although ocular motility was otherwise unrestricted with no nystagmus or strabismus. Brain MRI demonstrated the characteristic "molar tooth sign" with cerebellar vermis hypoplasia and thickened superior cerebellar peduncles, leading to a diagnosis of Joubert syndrome. The patient was referred for multidisciplinary management, including genetic testing, physical therapy, and genetic counseling. This case emphasizes that in pediatric patients presenting with COMA, comprehensive evaluation, including neuroimaging and developmental assessment, is essential, as isolated ophthalmological assessment may delay the diagnosis of underlying neurological conditions such as Joubert syndrome. Early identification enables appropriate management, surveillance for systemic complications, and timely genetic counseling, ultimately improving patient outcomes.

## Introduction

Congenital ocular motor apraxia (COMA) is a rare neurological disorder first described by Cogan in 1952 [1]. It has as its main symptom an inability to initiate horizontal voluntary eye movements (saccades). Patients exhibit a characteristic head movement called "head thrust" to look at an object. This is a compensatory behavior that uses the vestibulo-ocular reflex to move the eyes [1]. MRI may show abnormalities such as cerebellar worm hypoplasia and enlarged fourth ventricle, but may be normal [2]. About half of the patients have concomitant cognitive developmental disorders such as learning disabilities and intellectual disabilities, and motor and language developmental delays are also frequently observed [3]. In addition, COMA is often associated with Joubert's syndrome (JS), which is not simply an eye movement abnormality but is often a manifestation of a broader neurodevelopmental disorder [1-3]. Therefore, COMA may represent the first clinical clue of underlying JS, and recognition of this sign is critical for early diagnosis and appropriate management.

JS is a rare autosomal recessive neurodevelopmental disorder characterized by a distinctive “molar tooth sign” on brain MRI, with an estimated incidence of approximately one in 100,000 live births [4,5]. The primary clinical features of this disorder include progressive ataxia with muscle hypotonia from infancy, generalized developmental delay, oculomotor apraxia, and abnormal breathing patterns [6]. The “molar tooth sign” is a characteristic finding observed on axial MRI slices of the brain, characterized by a combination of hypoplasia of the cerebellar vermis, horizontal thickening and elongation of the superior cerebellar peduncles, and abnormally deep interpeduncular fossae, giving the appearance of molar teeth [7]. This finding is diagnostic of JS.

We report a case of COMA suspected to be related to JS. This case report underscores the importance of multidisciplinary collaboration in the evaluation of pediatric patients with congenital oculomotor palsy and emphasizes the need for brain imaging and developmental assessment, as well as thorough fundus examination to identify underlying conditions such as JS.

## Case presentation

A nine-month-old female infant was referred to our hospital for evaluation of developmental delay and abnormal eye movements. She was born at term after an uneventful pregnancy and delivery, with a birth weight of 2,800 grams. There was no significant family history of neurological or genetic disorders.

At presentation, the patient exhibited marked motor developmental delay. She was unable to roll over or sit without support. Neurological examination revealed increased muscle tone (hypertonia), truncal arching, and poor head control. Notably, she demonstrated oculomotor apraxia, characterized by an inability to initiate voluntary saccadic eye movements, requiring head thrusts to compensate for gaze shifts (Video 1).

**Video 1:** Head thrust maneuver observed in the patient A head thrust was observed when the patient attempted to fixate on an object positioned to the right. Specifically, after turning her face to the left, the patient rapidly moved her head to align her gaze with the target on the right. Similarly, a head thrust was evident when fixating on a target positioned to the left following a rightward head turn. Written and signed permission was taken from the patient's parents/legal guardians (in both English and Japanese) for publication of this video in an open-access, online journal.

**Figure 1:**
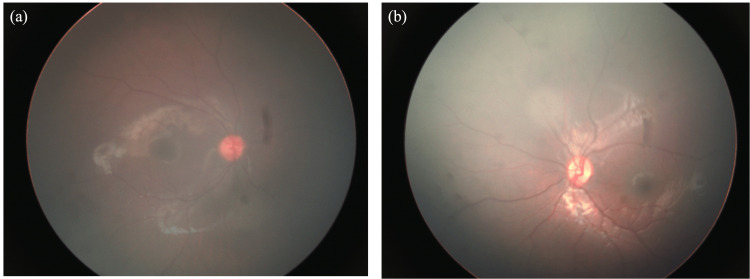
Fundus images at initial examination (a) right eye, (b) left eye

Ophthalmological assessment showed no limitation of ocular motility, but a positive head thrust test was observed (Figure 1). Cycloplegic refraction with tropicamide revealed bilateral hyperopia with astigmatism (right eye: +4.00 -0.75 × 75°; left eye: +3.25 -0.75 × 16°).

**Figure 2:**
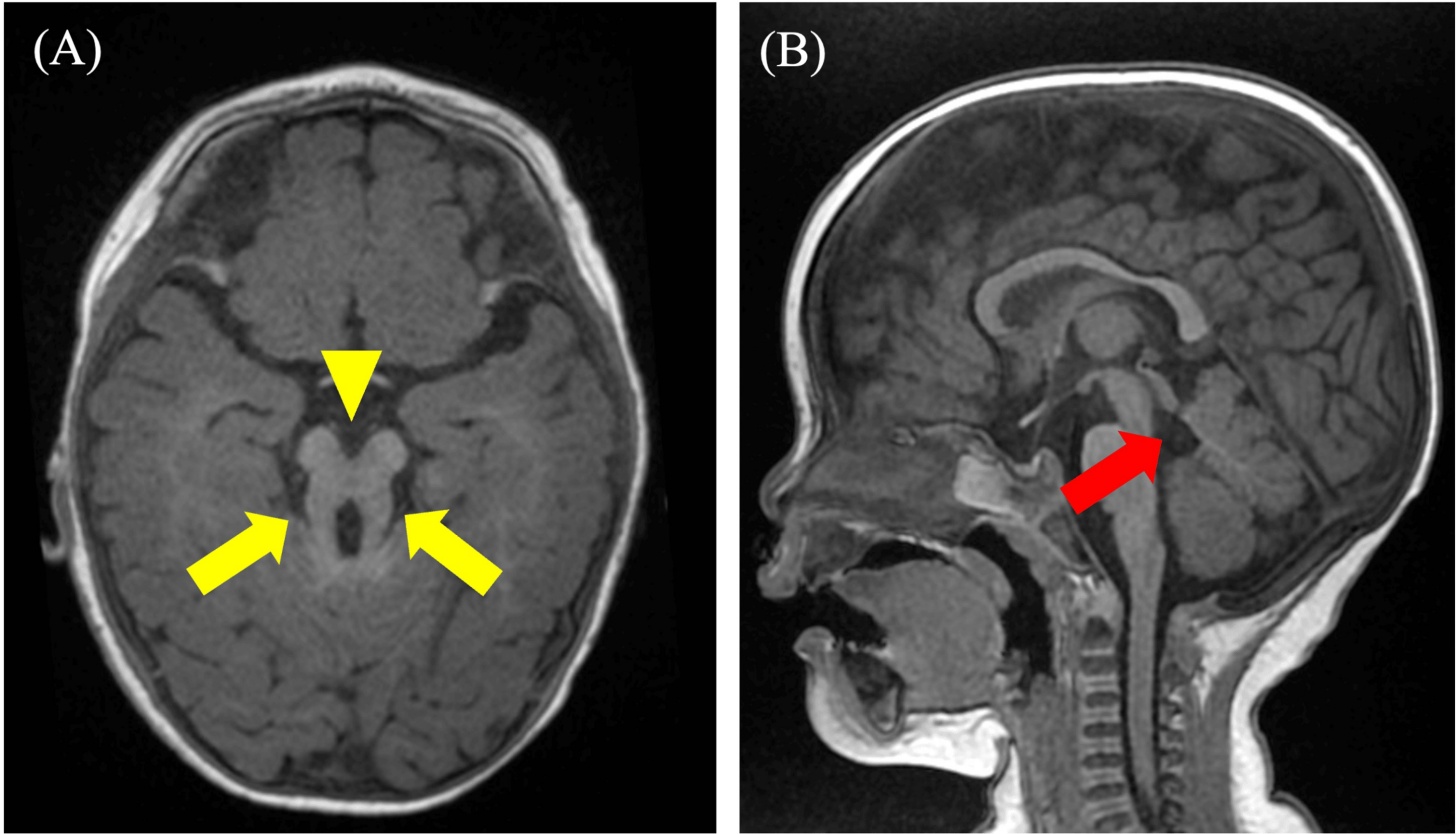
Non-contrast MRI of the head, (A) horizontal section and (B) sagittal section. The molar tooth sign is observed, consisting of a thick, straight superior cerebellar peduncle (yellow arrow), a deep interpeduncular fossa (yellow arrowhead), and hypoplasia of the cerebellar vermis (red arrow).

A magnetic resonance imaging (MRI) of the brain and a full-body examination from the pediatric department were performed. MRI revealed the characteristic "molar tooth sign," consisting of thickened and straightened superior cerebellar peduncles and hypoplasia of the cerebellar vermis (Figure 2). These findings were suggestive of JS.

Laboratory investigations, including blood and urine analyses, were unremarkable. Abdominal ultrasonography revealed no hepatic or renal abnormalities. Based on the clinical presentation and neuroimaging findings, a diagnosis of JS was strongly suspected. The patient was referred to a multidisciplinary team, including pediatric neurology and ophthalmology, for comprehensive management.

Following the diagnosis, the following interventions were initiated: Genetic testing was considered to confirm the diagnosis and identify the specific genetic mutation, physical therapy was started to address motor developmental delay and hypertonia, occupational therapy was initiated to improve trunk control and functional abilities, regular ophthalmological follow-up was scheduled to monitor visual development, comprehensive screening for associated complications was performed, including renal ultrasound and hepatic function tests, which showed no abnormalities at the time of evaluation, and the family was educated about developmental expectations and the importance of ongoing multidisciplinary surveillance.

At the ophthalmology examination at the age of one year and nine months, ocular motor apraxia persisted, but fixation and pursuit were good. Regarding motor and language development at the age of one year 11 months, the child could stand while holding onto something, but could not stand independently. Meaningful words were not yet recognized, but no regression was observed. At the most recent follow-up at the age of two years and three months, the patient continued to show gradual developmental progress with ongoing therapeutic interventions.

## Discussion

This case highlights several important clinical and diagnostic considerations regarding congenital ocular motor apraxia as an early manifestation of JS.

Our patient's presentation aligns with previous reports demonstrating that COMA can serve as an initial sign of underlying ciliopathies. Wente et al. reported that approximately 52% of patients with COMA had the molar tooth sign diagnostic for JS, and 57% showed cerebellar vermian abnormalities [3]. Similarly, our patient exhibited the characteristic compensatory head thrust maneuver described by Schroder et al., which is characteristic of COMA [1]. However, unlike isolated COMA cases reported in the literature, our patient presented with significant motor developmental delay and hypertonia at nine months of age, which raised immediate suspicion for a broader neurodevelopmental disorder [2].

The neuroimaging findings in our case are consistent with the classic description of JS by Romani et al., who emphasized the diagnostic value of the "molar tooth sign" on brain MRI [4]. This radiological hallmark, characterized by thickened superior cerebellar peduncles and vermian hypoplasia, was present in our patient and proved pivotal in establishing the diagnosis. Poretti et al. analyzed 75 JS patients and found that while the molar tooth sign is nearly universal, the degree of vermian hypoplasia and associated supratentorial abnormalities can vary considerably [8]. Our patient showed moderate vermian hypoplasia without significant supratentorial involvement, placing her within the more common phenotypic presentation.

An interesting contrast between our case and typically reported cases relates to muscle tone abnormalities. While classic JS is characterized by early hypotonia that is observed in nearly all patients, our patient demonstrated hypertonia with truncal arching at nine months of age [4,9]. This atypical presentation underscores the phenotypic variability of JS and emphasizes that the diagnosis should not be excluded based on atypical muscle tone findings.

Furthermore, our patient did not exhibit nystagmus at presentation, which is reported in approximately 72% of JS cases according to Wang et al. [10]. The absence of nystagmus in our case may be age-related, as some ocular features of JS can evolve over time, or may represent a milder ocular phenotype. This highlights the importance of longitudinal ophthalmological surveillance even when classic ocular findings are not initially present.

The diagnostic journey in our case emphasizes several key principles. First, the presence of COMA should prompt comprehensive neuroimaging rather than isolated ophthalmological evaluation. Sargent et al. demonstrated that approximately 60% of COMA patients have abnormal brain MRI findings, with cerebellar abnormalities being most common [2]. This finding supports the recommendation for routine brain MRI in all patients presenting with COMA, regardless of whether other neurological signs are evident.

Second, the multidisciplinary approach proved essential in our case. Collaboration with pediatric neurology facilitated a comprehensive developmental assessment and recognition of motor delays that extended beyond the ocular manifestations. Gonzalez-Gordillo et al. emphasized that early diagnosis of JS, ideally in the neonatal or early infantile period, allows for timely intervention and family counseling [11]. In our case, diagnosis at nine months enabled prompt initiation of physical and occupational therapy, which are crucial for optimizing motor outcomes in JS patients.

Brancati et al. reported that after the first months of life, global prognosis varies considerably among JS-related disorders subgroups, depending on the extent and severity of organ involvement, with renal and hepatic complications representing the major determinants of long-term outcome [9]. In our patient, initial screening for renal and hepatic involvement was negative, but continued monitoring will be essential. The early identification of JS in our case allows for anticipatory guidance and proactive screening for these potential complications.

JS is inherited in an autosomal recessive manner, with mutations in over 35 different genes identified to date [4,6]. Genetic testing for our patients is currently under consideration. If implemented, it is expected to provide valuable information for family counseling and recurrence risk assessment. Parisi et al. emphasized that molecular diagnosis can also inform prognosis to some extent, as certain genetic subtypes are associated with more severe organ involvement [6]. Additionally, genetic confirmation will enable prenatal diagnosis in future pregnancies.

One limitation of our case is the relatively short follow-up period at the time of this report. Longer-term follow-up will be necessary to fully characterize our patient's developmental trajectory and to monitor for potential systemic complications. Additionally, the final genetic results are pending and will provide important information for phenotype-genotype correlation.

The key learning points from this case are: (i) COMA should be considered a red flag for underlying neurological disorders, particularly JS, (ii) brain MRI is essential in the evaluation of all COMA patients, (iii) atypical features such as hypertonia should not exclude the diagnosis of JS, (iv) a multidisciplinary approach is crucial for comprehensive diagnosis and management, and (v) early identification enables appropriate therapeutic interventions and family counseling.

## Conclusions

This case reinforces the critical importance of recognizing COMA as a potential harbinger of JS and other neurodevelopmental disorders. Our patient's presentation, characterized by COMA with atypical hypertonia, illustrates the phenotypic variability of JS and underscores the need for comprehensive evaluation, including brain MRI and developmental assessment. Early diagnosis through multidisciplinary collaboration enables timely intervention, appropriate surveillance for systemic complications, and informed genetic counseling for affected families. This case adds to the growing body of literature emphasizing that isolated ophthalmological assessment is insufficient in children presenting with COMA, and that a holistic, collaborative approach is essential for optimal patient care.
